# Smartphone-measured tremor and tacrolimus trough levels in kidney transplant recipients: an exploratory prospective study

**DOI:** 10.3389/frtra.2026.1870210

**Published:** 2026-07-09

**Authors:** Annudesh Liyanage, Caitlin Schneider, Julien Renard, Rohit Singla, Jorden Hetherington, Steven Tang, Ted Hoyda, James Lan, Cindy Luo, David Harriman, Christopher Nguan

**Affiliations:** 1 Faculty of Medicine, University of British Columbia, Vancouver, BC, Canada; 2 Department of Electrical and Computer Engineering, University of British Columbia, Vancouver, BC, Canada; 3 Department of Urologic Sciences, University of British Columbia, Vancouver, BC, Canada; 4 School of Biomedical Engineering, University of British Columbia, Vancouver, BC, Canada; 5 Department of Nephrology, Vancouver General Hospital, Vancouver, BC, Canada

**Keywords:** digital biomarkers, kidney transplantation, machine learning, tacrolimus, tremor analysis

## Abstract

Tacrolimus commonly causes tremor as a side effect, although the relationship between tremor and tacrolimus drug levels is not well established. This work presents the development of a smartphone-based tremor assessment to explore whether tremor features are associated with tacrolimus trough concentrations, including whether individualized models show promise in tremor-sensitive kidney transplant patients. Using smartphone accelerometers, an app was created to record resting and postural tremors in transplant recipients during routine follow-up visits. Tacrolimus trough concentration and doses were recorded at each visit. We evaluated associations between tremor features and tacrolimus trough levels and, in exploratory analyses, assessed regression models both at the cohort level and within individuals with sufficient longitudinal data. Sixty-nine kidney transplant recipients were included. Correlations were found between self-reported tremor severity and tremor features, but no significant association was observed between tacrolimus trough concentration and tremor features at the cohort level (*ρ* = 0.022, *p* = 0.447). Population-level regression models showed poor predictive performance. *post-hoc* exploratory individualized models among patients with adequate longitudinal data (*n* = 8) provided an averaged root mean squared error of 2.33 ± 1.42 µg/L; three patients achieved root mean squared errors below 1.3 µg/L. Smartphone tremor features did not support population-level prediction of tacrolimus trough concentrations in kidney transplant recipients. Exploratory individualized regression models yielded potentially useful performance in a small subset; consistent with heterogeneity in tacrolimus tremor severity. These findings are hypothesis-generating; larger prospective studies should focus on identifying and validating tremor-sensitive patients for individualized approaches.

## 1 Introduction

Chronic kidney disease is a global epidemic with roughly 850 million people affected worldwide as of 2022 (1). Many progress to end stage renal disease (ESRD) and eventually require renal replacement therapy. Compared to other treatment modalities, kidney transplantation yields superior outcomes in quality and duration of life, as well as cost-effectiveness to the health care system (2).

Following transplantation, patients require immunosuppressant therapy to prevent allograft rejection. Tacrolimus is a widely used primary immunosuppressive agent prescribed to an estimated 93.5% of renal transplant recipients (3, 4). Importantly, tacrolimus has shown to be a pillar of immunosuppression in reducing transplant rejection, preserving renal function and ensuring cost-effectiveness compared to alternatives (5–7).

A major challenge with tacrolimus remains its narrow therapeutic window of 5–20 µg/L though many programs use a more conservative 5–15 µg/L window instead (8, 9). These levels suggest that the minimum threshold for drug efficacy is 10 µg/L lower than the upper-bound to avoid toxic side effects. As a result, frequent blood tests measuring tacrolimus trough levels, often daily in the first month, are required (3). This poses practical issues for recipients as specialized centres equipped for serum tacrolimus trough testing may not be equitably accessible to all, particularly for long term post-transplantation in a geographically distributed program.

Tacrolimus is associated with well-known side effects including nephrotoxicity, hypertension, dyslipidemia and neurotoxicity (8). Neurotoxic tremor is very common with up to half of users experiencing this side effect. This tremor usually presents as a symmetric action tremor, induced by voluntary muscle contraction, and possibly as a resting tremor (10). Most side effects of tacrolimus improve or resolve with reduced dosing or a switch to an alternative immunosuppressant. Reliable and regular tacrolimus trough level monitoring remains a mainstay in the long-term strategy to avoid toxic side effects while ensuring adequate immunosuppression. However, the presence and severity of tacrolimus-associated tremor vary substantially between individuals, with only a subset of recipients demonstrating clinically appreciable symptoms despite comparable drug exposure. This inter-individual variability suggests that tremor may reflect differential biological sensitivity to tacrolimus rather than a uniform dose-dependent event.

Therefore, there is a need to explore non-invasive monitoring approaches that may complement existing methods for tacrolimus drug level monitoring. Doing so will reduce testing costs, prevent additional risks from invasive techniques, facilitate agile dosage adjustments in the periphery, and improve side effect management with more frequent monitoring. Prior to creating such tools, further investigation into digital tremor phenotyping and tacrolimus-associated neurotoxicity is necessary.

Accordingly, approaches that assume a consistent population-level relationship between tremor characteristics and tacrolimus exposure may be inherently limited. Smartphone applications have shown utility in recording and analysing tremor signals, enabling quantitative characterization of tremor features in clinical settings (11). Additionally, tremor models have been developed for providing quantitative measures of tremor signals. This includes autoregressive models of order two for modelling physiological tremors and weighted-frequency Fourier linear combiner to model pathological tremors (12). More recently, to capture the non-linear relationship between tremor parameters and clinical variables, machine learning techniques have been proposed. These have been used for both classification and regression problems with traditional parameters, such as peak frequency, area under time-series curve, and full width at half maximum, used to make predictions (13). An improved understanding of whether tremor signals contain individualized, biologically meaningful information is therefore required before such approaches can be translated into clinical monitoring tools.

Therefore, we developed a smartphone application, leveraging the built-in three-axis accelerometer, to record tremor characteristics in kidney transplant recipients prescribed tacrolimus to investigate associations between smartphone-measured tremor features and tacrolimus trough concentrations. Overall, this is an exploratory study of digital tremor phenotyping and tacrolimus-associated tremor with a focus on evaluating the potential for modelling these relationships at both the population and individual levels.

## 2 Materials and methods

### 2.1 Patient enrolment

A single-center observational cohort study with *post hoc* exploratory modeling was conducted recruiting renal transplant recipients at a major transplant center (Vancouver General Hospital, Vancouver, Canada) under the appropriate research ethics board (H17-02564). Informed consent was obtained from all recipients. The inclusion criteria encompassed all renal transplant recipients presenting for transplantation between the ages of 18 to 65, capable of consent, and prescribed tacrolimus as part of an immunosuppression regimen. Patients with a different organ transplant type, or prescribed alternate primary immunosuppressants, medications that interfere with tacrolimus, or medications that impact tremor were excluded. Visits occurred between February 2018 to March 2020.

### 2.2 Study design

**Figure 1:**
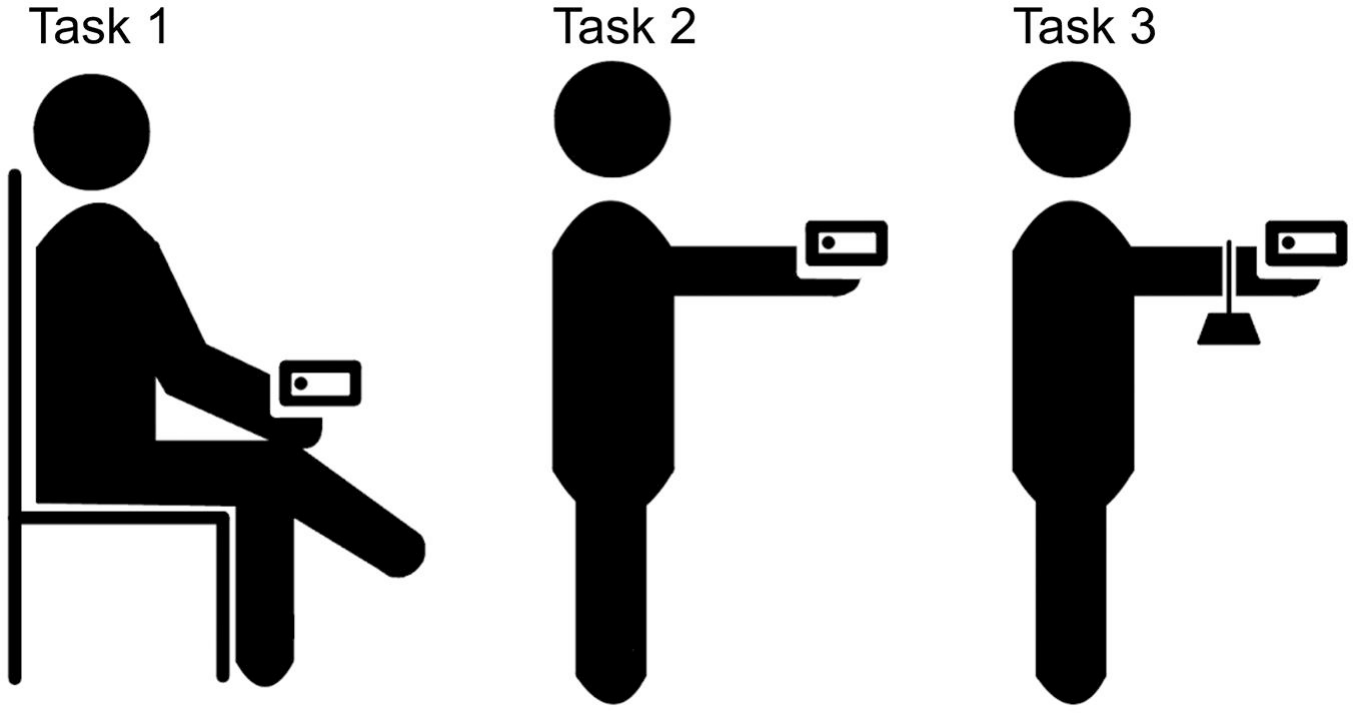
Images of tremor tasks. Task 1 with the phone held in hand and resting on the patient's leg (resting tremor). Task 2 with the phone held flat with the arm outstretched in front of the patient and Task 3 is the same set-up as Task 2 except that the arm has a 500 g wrist weight (postural tremor).

The clinic visits preceding transplantation registered patient demographic data including age, gender, handedness, height, weight, creatinine and self-reported tremor severity baseline. Subsequent post-transplant clinic visits also included daily tacrolimus dosage and morning pre-dose tacrolimus trough levels. Of note, the self-reported tremor severity was graded by the patient on a scale of 0 (no tremor experienced) to 9 (maximally bothersome tremor). The patient performed tremor tasks (Figure 1) at each clinic visit. Post-transplant follow-up visits occurred every second day for the first eight weeks post-transplant, followed by once a week for a month and then once every two weeks until discharged to home programs. More frequent visits were necessary earlier in the post-transplant period as this was when there was the most variance in drug dosage. Tacrolimus trough level was an objective outcome and therefore no blinding was required.

A custom coded Android application running on a OnePlus Nord N-series smartphone was used to record three-axis accelerometer data, sampled at 100 Hz. This is known to have an accelerometer range between ±2 g to 4 g (where g is acceleration due to gravity). Basic smartphone accelerometers have been found to be sufficiently sensitive to capture low-amplitude movement, which is the case for tremors, but their absolute precision is limited (14). With the phone held in the patient's hand, a resting tremor was recorded while the hand was supported in the patient's lap (Task 1); a postural action tremor while the arm was flat and fully outstretched (Task 2) and repeated with a 500 g wrist weight (Task 3). This is summarized with corresponding task numbers in Figure 1. Each of these tasks were repeated with both hands for twenty seconds. Clinical data and tremor parameters are found in Supplementary Data Sheet S1.

### 2.3 Data analysis

All analyses were conducted in an exploratory framework to assess whether tremor-derived features contain information related to tacrolimus exposure, rather than to develop or validate a clinically deployable predictive model. For such analyses, all cases with a tacrolimus trough level of 0 µg/L were excluded as this may have distorted relationship between tremor features and tacrolimus concentrations. Two complementary analytical approaches were used. The first utilized signal processing methods to assess for the presence of univariate associations between any tremor parameter and tacrolimus trough levels. An autoregressive moving average model (ARMA) of order (2, q), with the q selected based on the Akaike information criteria (AIC), was developed. The residuals were calculated from the model, and the Ljung-Box Q-test was used to assess for any unexplained correlation. This model was then used to assess correlations between tremor parameters with tremor severity and tacrolimus trough levels. Specifically, we assessed for correlations between the ARMA model parameters (i.e., the autoregressively determined coefficients) and the calculated variance and peak frequency with tremor severity and tacrolimus trough levels (15, 16). These were quantified using a Spearman rank test for ranked tremor severity and Pearson's test for continuous variables assuming statistical significance with a *p*-value less than 0.05. Given the exploratory nature of these analyses and the multiple comparisons performed across tasks, axes, and hands, no formal correction for multiplicity was applied; significant associations are therefore interpreted as exploratory and hypothesis-generating rather than confirmatory. Of note, higher tremor severities (7–9) were excluded from correlation calculations due to limited sample sizes. The dataset was also separated based on metadata, such as age, gender and handedness, while assessing for any correlations.

The second approach used machine learning models applied as exploratory tools to evaluate potential multivariate relationships between tremor features and tacrolimus trough levels. A root mean squared error (RMSE) of 1.3 µg/L was determined to be clinically acceptable based on consultation with our transplant pharmacist and supported by related tacrolimus prediction studies (17). This threshold was chosen to reflect the degree of prediction error that would be unlikely to alter expected therapeutic decision thresholds. Tacrolimus trough concentrations are subject to both analytical assay variability and biological variability (18). Based on input from our transplant pharmacology team, a prediction error of 1.3 µg/L was considered sufficient to remain in keeping with variability encountered during routine monitoring. This error would unlikely result in different clinical management. Additionally, we defined an RMSE below 2 µg/L as borderline, and above 2 µg/L as inadequate.

Key variables included the median and peak frequencies, peak and total power, area under the frequency curve, full width at half maximum and entropy. Visits with missing clinical information were included since the model could address this, but visits with missing tremor records or tacrolimus levels were excluded. To assess population-level relationships, five models were trained on the full dataset using nested cross-validation, with an inner loop for hyperparameter tuning by grid search (Supplementary Methods) and an outer loop for performance estimation. The model architectures used included eXtreme gradient Boosting, CatBoost, Random Forest, linear regression and support vector machine. The RMSE from the model with lowest error is provided along with the prediction and residual plots.

Given anticipated inter-individual variability, additional analyses were conducted at the individual patient level to explore whether patient-specific relationships could be identified in those with sufficient longitudinal data. Subsequently, patient-specific models were trained on individual patient data. Patients were selected based on having a sufficient number of longitudinal observations to enable within-patient relationships. To train models with at least two parameters, roughly 20 data points is required. However, our study population had too few patients that met this threshold. Therefore, a cutoff of 17 data points was chosen which included eight patients. However, thresholds of 20 and 15 data points were evaluated as well. Models were trained using (n-3) data points with the final 3 saved for the test set. For clinical variables, only daily dosage (and not gender or age) was used. The RMSE was calculated for all cases. Given the limited sample size, analyses were not designed for external validation and should be interpreted as exploratory. Additional technical details for the data analysis can be found in the Supplementary Methods section. A completed TRIPOD checklist is attached with the paper (Supplementary Table S1).

## 3 Results

### 3.1 Patient characteristics

**Figure 2:**
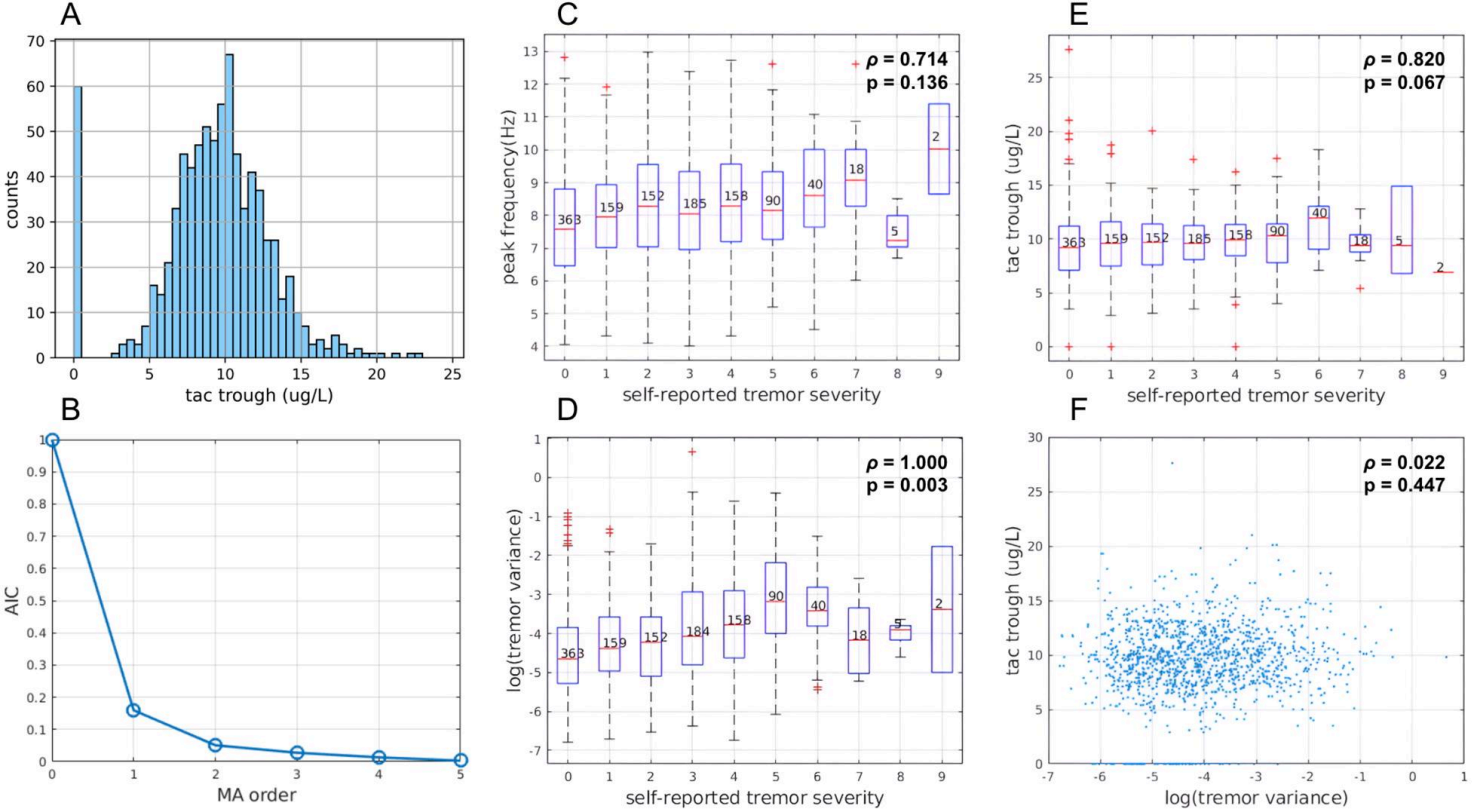
Signal processing analysis results. **(A)** Distribution of tacrolimus trough levels in the study population. This includes measurements of all patients at all visits including pre-transplant (tacrolimus = 0). **(B)** ARMA model selection based on the AIC. The AIC is normalized and averaged over the whole dataset. **(C)** Box plot of the tremor signal peak frequency as a function of the self-reported tremor severity. The sample size for each box is displayed. *ρ* represents the correlation coefficient (Spearman rank test for categorical independent variable, Pearson test for continuous independent variable) and *p* represents the associated *p*-value. **(D)** Box plot of the logarithm of tremor signal variance as a function of the self-reported tremor severity. **(E)** Box plot of tacrolimus trough concentration as a function of the self-reported tremor severity. **(F)** Scatter plot of the tacrolimus trough level as a function of the logarithm of tremor variance. **(C–F)** are examples for Task 1, z-axis, and averaged over both hands.

A total of sixty-nine kidney transplant recipients were included in this study. Demographic variables are provided in Table 1. The tacrolimus trough levels were mainly concentrated in the 5–15 µg/L range (therapeutic range, 687 cases) with few cases in the subtherapeutic (<5 µg/L, 66 cases) and toxic ranges (>15 µg/L, 31 cases) ranges (Figure 2A).

**Table 1:** Baseline patient characteristics.

Characteristic	Values
n	69
Age, years	51.7 (12.3)
Gender, male	48 (69.6)
Height, cm	171.7 (11.4)
Mean weight, kg	78.9 (18.9)
Number of visits	11.4 (4.8)
Duration of follow-up, days	99.2 (46.8)
Tacrolimus daily dose, mg/day	9.7 (4.1)
Tacrolimus trough, µg/L	9.2 (2.2)
Creatinine, umol/L	195.6 (122.4)
Tremor severity, [0–9]	1.9 (1.5)

Values are recorded as [mean (one standard deviation)] for all variables except for gender which is reported as [counts (frequency)]. Weight, tacrolimus daily dose, tacrolimus trough and creatinine are averaged over all visits and then averaged over patients to provide values reported in the table. This data is provided in full in Supplementary Data Sheet S1.

### 3.2 Signal processing approach

The AIC was used to select the q parameter for the ARMA(2,q) model (Figure 2B). The elbow in the curve indicated that *q* = 1 was the optimal candidate and therefore, the ARMA(2,1) model was selected. Compared to harmonic models, the residuals from fitting the ARMA(2,1) model were much closer to a gaussian distribution. However, the distribution still exhibited a heavier tail, and the residuals failed the Ljung-Box Q-test that test for a correlation unexplained by the model.

Using task 1 data along the z-axis, averaged over both hands, correlations were assessed between self-reported tremor severity, tremor parameters, and tacrolimus trough levels. For the logarithm of tremor variance, there was a monotonic relationship with self-reported tremor severity (*ρ* = 1.000, *p* = 0.003, Figure 2D). For peak frequency, there was a correlation but this did not reach statistical significance (*ρ* = 0.714, *p* = 0.136, Figure 2C). Similar conclusions were obtained for other accelerometer axes and tasks performed.

Self-reported tremor severity and tacrolimus trough levels did show some correlation but again, this did not reach statistical significance (*ρ* = 0.820, *p* = 0.067, Figure 2E). No statistically significant associations were found between ARMA model parameters, peak frequency and tremor variance with tacrolimus trough level. For instance, the correlation between the logarithm of tremor variance and tacrolimus trough level did not demonstrate evidence of correlation (*ρ* = 0.022, *p* = 0.447, Figure 2F). Furthermore, separating the dataset based on other metadata did not modify this result and these results were similar across all tasks and axes. No linear correlation was found between any tremor signal parameters and tacrolimus trough levels.

### 3.3 Machine learning approach

**Figure 3:**
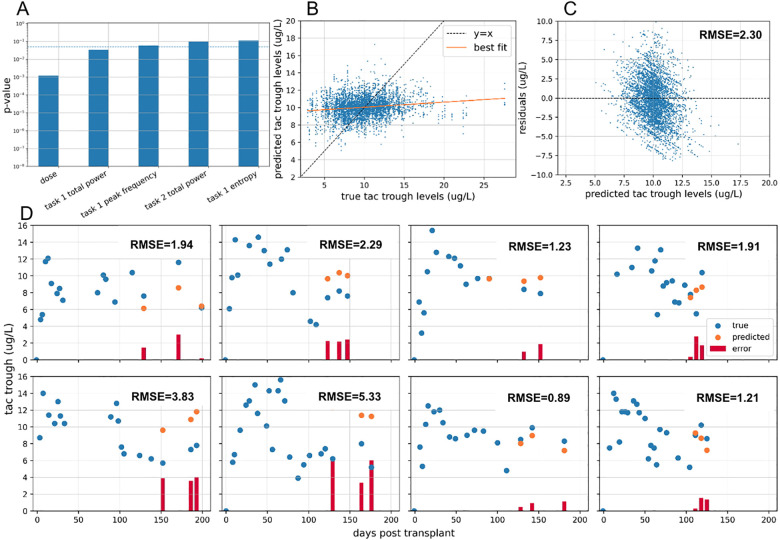
Machine learning random forest model analysis results. **(A)** Selected model variables *p*-values (all dominant hand) used in regression model with dashed blue line showing *p*-value = 0.05. **(B)** True and predicted tacrolimus trough levels comparison with black line representing unity and the orange line representing the best-fit. **(C)** Residuals plot with residual = true-predicted and black line represents *y* = 0 and the root mean squared error is reported as 2.30 ± 1.95 µg/L. **(D)** Tacrolimus trough level predictions from Random Forest regressor trained on individual patient data. The eight subplots each represent a unique patient. Blue dots are the true tacrolimus trough levels, and the orange dots are predicted trough levels for the final three data points. The red bars provide the absolute error, and the root mean squared error is reported for each case. Across the eight cases, the averaged root mean squared error was 2.33 ± 1.42 µg/L.

Among the evaluated models for population-level analysis, the Random Forest approach demonstrated the lowest error within this analytical framework. The selected variables were the tacrolimus dose, task 1 dominant hand total power, peak frequency, entropy and task 2 dominant hand total power. The predictions from this model and its associated residual values are provided in Figures 3B,C. The model achieved a clinically unacceptable RMSE of 2.30 ± 1.95 µg/L. Predictions were narrowly distributed in the 7.5–12.5 µg/L range.

For individualized regression models, eight patients with at least 17 data points were used to train a Random Forest regression model using (n-3) data points with the final 3 saved for the test set. ANOVA determined two to three statistically significant predictors variables for each individual model. The averaged RMSE across the eight patients was 2.33 ± 1.42 µg/L. The predictions are plotted against true values in Figure 3D. Three of eight patients achieved a potentially promising RMSE (<1.3 µg/L). When using a data point threshold of 20, only two cases met this threshold and both had an RMSE below 1.3 µg/L (1.21 and 0.89 µg/L). With a threshold of 15, a total of 15 cases were included and achieved a mean RMSE of 2.51 ± 1.18 µg/L. Additionally, only four of the 15 cases achieved an RMSE below 1.3 µg/L.

## 4 Discussion

We evaluated whether smartphone-based tremor analysis captures signal related to tacrolimus exposure and assessed its feasibility as a non-invasive monitoring approach in renal transplant recipients. To our knowledge, this is the first exploratory study of its kind. While earlier studies have characterized tacrolimus-associated tremor using clinician-rated scales or laboratory-grade accelerometers (19), few have explored this with smartphone applications. Prior studies have used tremor analysis in other areas, such as predicting Parkinson's disease symptoms and medication effectiveness (20). By integrating built-in smartphone sensors with our described models, this work presents a novel approach to non-invasive assessment of tacrolimus-associated tremor. Our findings indicate that tremor-based approaches are not generalizable at the population level due to varying tremor sensitivities. However, for putative tremor-responsive patients, there may be potential for application within a subset of patients, warranting further investigation.

Previous studies have explained that only 34%–54% of kidney transplant recipients develop a tacrolimus-associated tremor (21). Our results demonstrate that neither univariate associations nor multivariate modelling approaches identified meaningful relationships at the population level. This likely reflects substantial inter-individual variability in tacrolimus-associated tremor expression. Furthermore, this may not necessarily exclude a relationship between tacrolimus exposure and tremor biology as this may indicate that tacrolimus neurotoxicity is driven by aspects of drug exposure not captured by trough monitoring, including peak concentrations or cumulative exposure. Nonetheless, in the context of following trough concentrations, these findings suggest that future work should prioritize identifying and characterizing putative tremor-responsive patients rather than applying uniform models across all recipients.

Efforts to formally isolate this subset were limited by small sample size (*n* = 8), which precluded meaningful subgroup analyses. However, exploratory individualized models achieved potentially promising performance in three of the eight patients. A similar proportion met the threshold when a cut-off of 15 data points was used (four of fifteen patients). Both proportions are consistent with what chance alone would produce: under a null success probability of 0.5, the binomial probability of observing exactly three of eight patients below the RMSE threshold is approximately 0.22, well above the conventional 0.05 significance level. These subset findings should therefore be interpreted as hypothesis-generating rather than as evidence of a reproducible effect. These findings suggest that tremor-derived features may contain individualized signal related to tacrolimus exposure in select patients. After identifying patients based on their tremor responsiveness to tacrolimus in the early post-transplant phase, patient-specific models could be trained following the identification of responsive individuals. Although this preliminary analysis is limited by the number of patients with sufficient longitudinal data, these results warrant further investigation to evaluate the feasibility of an individualized modelling approach.

There are several limitations associated with this study. Firstly, there is an inherent limitation of using tremor as a surrogate for tacrolimus trough levels considering that tremor is a toxic effect experienced at high levels. This issue could not be avoided as tacrolimus trough levels are the accepted measure for monitoring adequate dosing. However, tacrolimus-associated tremor may better relate to measures of peak drug exposures. Key factors such as peak concentration, area under the concentration-time curve (AUC), time since last dose, and tacrolimus formulation may influence tremor independently from tacrolimus trough levels. As a result, the absence of an association between smartphone-measured tremor and tacrolimus trough levels does not necessarily exclude a relation between tacrolimus exposure and tremor biology. This may instead reflect limitations of trough concentrations as a surrogate for capturing pharmacologic drivers of tremor. Further studies using AUC-based monitoring, peak exposures, and more detailed pharmacokinetic data may better characterize this relationship. Additionally, raw accelerometer and gyroscope data would have improved signal quality but due to issues with collecting gyroscopic data (samples being missed, etc.), this was not possible. With this approach, there could have been more nuanced aspects of the tremors that could have been captured. Additionally, patient tremors could have also been impacted by other factors, including stress, fatigue, caffeine, and concomitant medications such as corticosteroids. It should also be noted that in the signal processing approach, since the Ljung-Box test did fail, the ARMA-derived tremor variance and peak frequency may be biased and this may have precluded correlations from being identified. Furthermore, the population-level analyses treated repeated visits within patients as independent observations and did not model the resulting within-patient correlation, which may have influenced the population-level association estimates. Lastly, the limited number of data points prevents a definitive conclusion of the feasibility of individualized regression models. Population-level approaches may remain limited even with larger sample sizes due to underlying biological variability. However, for individualized models, larger datasets with sufficient longitudinal observations per patient will be required to more definitively assess feasibility.

In conclusion, in this exploratory and hypothesis-generating study, a novel smartphone application to measure tremor signals was assessed to explore the feasibility of non-invasive assessment of tacrolimus-associated tremor. The analysis of the tremor signals, using both signal processing and machine learning techniques, have demonstrated that a single population-level model was not sufficient to capture relationships between tacrolimus trough levels and tremor parameters. In other words, smartphone-derived tremor features did not support population-level trough prediction. This is attributable to heterogeneity in tremor sensitivity. For putative tremor-responsive patients, individualized models achieved potentially promising results in three of the eight patients. However, these findings are exploratory and limited by the number of patients with sufficient longitudinal follow-up precluding clinical implementation at this time. Such individualized approaches therefore remain hypothesis-generating. To further investigate the feasibility of tremor as a surrogate to infer tacrolimus trough levels in these putative tremor-responsive patients, this should be an avenue for future research.

## Data Availability

The datasets presented in this study can be found in online repositories. The names of the repository/repositories and accession number(s) can be found in the article/Supplementary Material.
