# Supplementary material for: Smartphone-measured tremor and tacrolimus trough levels in kidney transplant recipients: an exploratory prospective study
Source: Front Transplant. 2026 Jul 9;5:1870210. doi: 10.3389/frtra.2026.1870210 (PMC13391576; doi:10.3389/frtra.2026.1870210)
Supplement: Supplementary file 1 [file Supplementaryfile1.pdf]

Supplementary Material

1 Supplementary Methods

For both data analysis approaches, the application programming interface used to collect the accelerometer data was found to remove the gravity offset using a built-in adaptive filter which contaminated results, and as a result, a mitigation strategy was to use the last 10 seconds of accelerometer data which bypassed this limitation. Furthermore, all cases with a tacrolimus trough level of 0 ug/L were excluded and the range of tacrolimus trough levels in the study was observed to be [2.9 ug/L, 27.6 ug/L].

1.1 Signal Processing Approach

Visual analysis of tremor signals, in both time and frequency spectra, suggested that the tacrolimus induced tremor could be modelled as quasi periodic signals. Based on previous attempts to capture pathological tremors, a truncated harmonic series model using an autoregressive integrated moving average was tested (22). However, this model lacked a parameter to control signal amplitude which made accounting for the noise variance difficult between axes. For this reason, the amplitude was modelled with an autoregressive order 1 (AR(1)), indicating that only the previous value is used for future predictions, to modify the truncated harmonic series model. Hyperparameters were then estimated using Markov chain Monte Carlo methods: Metropolis-Hastings algorithm specifically (23). Due to the large number of parameters, this was very computationally expensive and therefore, simpler models were investigated.

Physiological tremors have been successfully modelled by AR(2) processes, which use two prior values to predict future points (12, 24). Due to the tacrolimus induced tremor being very mild or absent, we chose to fit an autoregressive moving average model (ARMA) of order 2 and of moving average order q (ARMA(2,q)). This q was selected using the Akaike information criterion (AIC). Model residuals were evaluated using the Ljung-Box Q-test to assess for remaining autocorrelation.

With the ARMA model fitted to the tremor signal, the power spectral density can be calculated and from this, tremor parameters can be calculated. Specifically, peak frequency and tremor variance are calculated using MATLAB's standard ARMA model methods using the power spectral density.

1.2 Machine Learning Approach

Tremors have been found to be a two-dimensional phenomenon as the oscillations occur around a relatively fixed axis in the direction of the forearm (25). Therefore, using principal component analysis, the data was reduced to its two main dimensions. From this point, recognizing that tremors involve an object with a fixed radius oscillating about a single axis, the data was converted to polar coordinates: (x,y) converted to (radius, theta). Only the theta angle was kept reducing the number of dimensions to one.

For time series analysis, the Hilbert transform was used to calculate the area under the curve, and the peak and mean amplitudes were measured as well. For frequency analysis, the time-series data was converted using the Fast Fourier Transform and, to account for all possible tremor types, filtered for

frequencies between 2 to 20 Hz (13). The power spectral density was obtained as well. From this, the median and peak frequencies, peak and total power, area under the frequency curve, full width at half maximum and entropy were calculated. Many of these terms are highly correlated but they were all calculated for ease in later steps of the analysis. Overall, this provided 88 tremor parameters (2 hands * 4 tasks * (3 time series parameters + 11 frequency parameters)) for each case of each patient along with the gender, age, daily dosage and tacrolimus trough level.

Models were trained using several machine learning models (eXtreme gradient Boosting, CatBoost, Random Forest, linear regression and support vector machine). Input and output variables were first standardized with a scaler. The input variables were filtered first using the variance inflation factor with a conservative threshold of 10 and then creating a correlation matrix and excluding one variable in any set (at random) that had a correlation factor greater than 0.7. The dataset was then split into training and test sets.

When training on the full dataset, a nested-cross validation was applied to the training data. This was done using patient-level clustered cross-validation for the outer loop. The inner loop was for hyperparameter tuning using a grid search strategy (Table 2) which only used training data. The outer loop was for feature selection (with inputs from the training set only) and model performance evaluation using the test set. Feature selection used analysis of variance (ANOVA) and selected variables with a p-value < 0.05.

When training models on individual patient data, feature selection (with inputs from the training set only) used ANOVA and significant variables were chosen. Hyperparameters were selected using the same strategy as above (Table 2) with only training data. Model performance metrics were assessed using a 10-fold cross validation strategy and averaged over 10 bootstraps. Model metrics were calculated using RMSE and used to compare models.

Parameter Name	Grid Search Space
Number of Estimators	[100, 200]
Maximum Depth	[None, 10, 20]
Minimum Samples Split	[2, 5]
Minimum Samples Leaf	[1, 2]

Table 2: Grid search for Random Forest regression model hyperparameter tuning.

2 Supplementary References

22. Bo APL, Poignet P, Geny C. Pathological tremor and voluntary motion modeling and online estimation for active compensation. IEEE Trans Neural Syst Rehabil Eng. 2011;19:177-85.
23. Ninness B, Henriksen S. Bayesian system identification via Markov chain Monte Carlo techniques. Automatica. 2010;46:40-51.
24. Timmer J. Modeling noisy time series: physiological tremor. Int J Bifurcation Chaos. 1998;8:1505-16.
25. Livi L, Rizzi R, Sadeghian A, Giuliani A, Vignatelli L, Chiaramonti R, et al. Discrimination and characterization of parkinsonian rest tremors by analyzing long-term correlations and multifractal signatures. IEEE Trans Biomed Eng. 2016;63:2243-9.
